# The evolution of research collaboration within and across disciplines in Italian Academia

**DOI:** 10.1007/s11192-016-2068-1

**Published:** 2016-07-15

**Authors:** Elisa Bellotti, Luka Kronegger, Luigi Guadalupi

**Affiliations:** 1 Department of Sociology, Mitchell Centre for Social Network Analysis, University of Manchester, Manchester, UK; 2 Faculty of Social Sciences, Centre for Methodology and Informatics, University of Ljubljana, Ljubljana, Slovenia; 3 Institute for Service Industry Research (IRAT), National Research Council (CNR), Rome, Italy

**Keywords:** Scientific networks, Cluster analysis, Interdisciplinary, Scientific collaborations, Social network analysis, Scientometrics

## Abstract

In sociology of science much attention is dedicated to the study of scientific networks, especially to co-authorship and citations in publications. Other trends of research have investigated the advantages, limits, performances and difficulties of interdisciplinary research, which is increasingly advocated by the main lines of public research funding. This paper explores the dynamics of interdisciplinary research in Italy over 10 years of scientific collaboration on research projects. Instead of looking at the output of research, i.e. publications, we analyse the original research proposals that have been funded by the Ministry of University and Research for a specific line of funding, the Research Projects of National Interest. In particular, we want to see how much interdisciplinary research has been conducted during the period under analysis and how changes in the overall amount of public funding might have affected disciplinary and interdisciplinary collaboration. We also want to cluster the similarities and differences of the amount of disciplinary and interdisciplinary collaboration across scientific disciplines, and see if it changes over time. Finally, we want to see if interdisciplinary projects receive an increasing share of funding compared to their disciplinary bounded counterparts. Our results indicate that while interdisciplinary research diminishes along the years, potentially responding to the contraction of public funding, research that cut across disciplinary boundaries overall receives more funding than research confined within disciplinary boundaries. Furthermore, the clustering procedure do not indicate clear and stable distinction between disciplines, but similar patterns of disciplinary and interdisciplinary collaboration are shown by discipline with common epistemological frameworks, which share compatible epistemologies of scientific investigations. We conclude by reflecting upon the implications of our findings for research policies and practices and by discussing future research in this area.

## Introduction

Social studies of science have long been interested in observing collaborations between researchers: departing from the idea that science is a simple accumulation of subsequent discoveries of objective truths, scholars have dug into the human negotiations that inform agreements, conflicts, and the social construction of scientific facts. A long and established stream of research investigates the role of social ties in scientific collaboration as key factors that shape the organization of scientific production. One way of analytically tackling the complexity of the structure of social relations is to adopt the methods and perspective of social network analysis. The approach is not new in this substantive area, as it dates back to the study of communication and citations in the search for what were initially called invisible colleges (Crane 1972), and to the study of the complexities of overlapping co-authorships and co-citations in the tradition of bibliometric and scientometric studies (De Solla Price 1965; Garfield 1955). Within this tradition researchers have attempted to map and visualize scientific collaboration in various ways (see Leydesdorff 1987 for a methodological review) as well as to cluster academic disciplines according to collaboration patters (Garfield 1955; Garfield et al. 1964; Kronegger et al. 2015), in order to observe the macro organization of scientific disciplines.

A further step in the study of the structure of science has focused on scientific collaborations that cut across traditional disciplines, and a fruitful debate has flourish around the definition of inter, trans, and multi-disciplinary projects (Bruce et al. 2004; Choi and Pak 2006; Jacobs and Frickel 2009). This focus is important because, as Jeffrey (2003: 539) said, “a central motivation for research funders to support studies that consider the contributions of more than one disciplinary field is the fact that real-world problems do not come in disciplinary-shaped boxes”. In this line of research studies range from the attempts to map the network of citations across the whole field of science (Boyack et al. 2005); to the identification of semantic themes common to multiple disciplines (Callon et al. 1983; Leydesdorff 1991); to small scale case studies that untangle the everyday practices of researchers involved in interdisciplinary projects (Cummings and Kiesler 2005; Barry et al. 2008; Hara et al. 2003; Jeffrey 2003; Porac et al. 2004).

As we will see in the first section of this paper, a popular approach to the study of research collaboration focuses on co-authorship data. Such an approach, although undoubtedly fruitful, presents some limits, insofar as it takes publications as the only proxy to measure research collaboration. The assumptions behind this common operationalization are (Subramanyam 1983):

1. That the number of papers produced is proportional to the research activity, as in all collaborations results in publications;
2. That all collaborators are mentioned in every publication;
3. That all co-authors have actually collaborated to the research activity;

There are several reasons why these assumptions may not be consistent with everyday scientific practices. First of all, publications may be rejected; therefore it is not straightforward that collaborations will result in publications (Katz and Martin 1997). Second, there might be cases in which collaborators are not mentioned: Laudel (2002), for example, reports that a large part of people involved in the preparation of a scientific paper do not appear as co-authors of the publication. Third, it can be the case that people named in an authors’ list may not have directly collaborated to the specific part of the project that has led to the publication, but are still included as part of the larger collaborative team (Cronin 2001), or because they are mentors or supervisors of young researchers who mention them for gratefulness or prestige. Finally, the practices of co-authorship are ruled by different social norms in different disciplines, as reflected by the distribution of numbers of co-authors (Mali et al. 2012; Kronegger et al. 2015), and by the different order in which they appear in an article (alphabetical order, main author first, main author last, etc., see Abramo et al. 2013). Thus, a complementary stream of research has developed that looks not only at the outputs of research, but also at the participation of scientists to successfully funded research projects (Breschi and Cusmano 2004; Protogerou et al. 2010), at the factors that facilitate or hamper such collaborations (Katz 1994; Maggioni and Uberti 2007; Scherngell and Barber 2011; Scherngell and Lata 2013), and at the relationship between the structure of collaborations and economic development (Maggioni et al. 2014).

This paper observes the dynamics of interdisciplinary research in Italy by looking at the scientific collaboration to research projects funded by the Ministry of University and Research (MIUR) from 2001 to 2010. Data have been gathered for a specific line of funding, the Research Projects of National Interest (PRIN) for all disciplines of Italian Academia. One of the advantages in using these kinds of data is that all the people mentioned in research projects are actual collaborators, given the fact we only consider projects that are successfully funded: once a researcher receives a grant, s/he has to comply at least with some of the tasks described in the original research proposal. One of the limits is that our data do not contain any information of the outcomes of such collaborations, for example on co-authored publications: therefore, like co-authorship studies, the analysis only offers a partial account of the collaboration process. Keeping this in mind, the goal of the paper is to describe the longitudinal patters of collaboration within the same discipline, and across different disciplines, in order to analyse the overall structure of disciplinary and interdisciplinary research in Italy, and how it changes over time. Furthermore, we want to link the patterns of disciplinary/interdisciplinary collaboration with the amount of money each discipline gained during the 10 years under analysis, to see if there is any difference in the share of funding for disciplinary and interdisciplinary projects.

Following this introduction, the first section of the paper reviews the literature on interdisciplinary science focusing on empirical research that have studied research collaboration, and on the definitions that have been advanced to systematize inter, trans, and multi-disciplinary cooperation. In the second section a brief overview of the organization of disciplines in Italian Academia, and of the PRIN line of funding during the 10 years under analysis is presented. We then define the research questions that are posed by the trends in MIUR funding for PRIN projects and that guide this article. The third section describes the dataset, the calculations that have been performed in order to analyse it, the analytical strategies that have been adopted to operationalise our research questions and the limits of the available data. Results are presented in the fourth section, which shows on one hand how interdisciplinary projects have declined for all disciplines, but on the other hand how successful research that cut across disciplinary boundaries has overall received more funding than research confined within disciplinary boundaries. Furthermore, we observe the similarity of disciplines in regards of their patterns of research collaboration, to explore the consistency of traditional classifications of science. The outcomes are discussed in the fifth section, while conclusions reflect upon the implications of our findings for research policies and practices and future research in this area.

## Collaboration in science: inter, trans, and multi-disciplinary research

The term discipline refers to the act of disciplining a branch of knowledge, of regulating the body of information produced by a scientific community which became necessary with the rapid expansion of scientific knowledge during the industrial revolution (Chubin 1976). But a discipline is also a “stable systemic community within which researchers concentrate their experience into a particular worldview” (Bruce et al. 2004: 458). Disciplines therefore include both ontological-epistemological and socio-historical dimensions (Kronegger et al. 2015). The first refers to the objects and methods of a speciality area (what is under study, and how it is studied), which are expected to be relatively stable over time, while the second stands for the more contingent institutional organization of knowledge (how scientific production is produced and accepted by its referral community, see Becher and Trowler 2001; Whitley 1984). The latter also guides the educational training of new scientists through academic curricula, and the distribution of funding across disciplines.

The stability of the ontological and epistemological dimension of a discipline has been firstly questioned by Kuhn’s theory of scientific revolutions (1970). According to Kuhn, science is dominated by paradigms, corpuses of shared beliefs among members of a speciality area where interests converge around a specific set of problems identified as significant for the advancements of knowledge. By supporting the same paradigm, researchers also share specific understandings of which research techniques are appropriate for investigating these problems and a sense of identity that is constructed via interpersonal networks and information sharing. It is in the interaction that happens in scientific communities that the ontological and epistemological aspects of a discipline are negotiated and eventually stabilized: the characteristics of stability and universality are thus not a precondition of science, but the results of the institutional organization of science. Following this logic, some scholars have proposed to distinguish between proper disciplines, which are in charge of the training of new scientists and form the teaching domain of science; and intellectual units, nested within and between disciplines, that comprise the research domain and can be defined as “scientific specialties” (Chubin 1976: 448). Similarly, Hagstrom (1970: 93) characterizes specialties as “microenvironments for research, as frequently traversed regions on a blurred map of science”, where scientists engage in similar research and intensively communicate with each other. This idea is also present in the studies of Crane (1972) and De Solla Price (1963) on invisible colleges, and in the ones of Kadushin (1966, 1968) on social circles.

According to these authors, it is in the core of specialities that scientific advancements are achieved, and the ontological and epistemological discussions take place. Scientists belonging to these cores exchange ideas and discuss their work more often with each other than with people outside their social circles. In social circles information easily circulates thanks to the chain or network of indirect interaction that connects most members to other members: members share common interests, which stimulate discussion and agreements as well as disagreements and conflicts (Kadushin 1968: 692). These negotiations are the social engine of scientific development, where scientific objects are discussed, contested, and eventually accepted to become solidified elements of a discipline, worth of being taught to new generations of scientists.

The idea that a speciality is structured as a network of social relations between its members has stimulated various attempts to operationalise and measure the shape and content of these networks. The key element in this process is to identify the relevant type of relationships that may link scientists. Social scientists have thus focused their attention to the various forms of interactions between researchers, looking at both formal and informal relationships. Informal relations, as in the type of interactions that may occur in the daily tasks of scientific work, have been the core of micro and qualitative studies, like the investigations of the Edinburgh and the Bath School (Barnes and Bloor 1982; Collins 1985) and of Laboratory studies (Gilbert and Mulkay 1984; Knorr-Cetina 1992) Formal relations have been mainly measured by using co-authorship of scientific articles or through citations, where scientists build their own work on each other’s’ shoulder, and the analysis of co-authorship and citations (or co-citations) have long dominated bibliometric and scientometric studies. A full review of this type of literature is not possible here, and it is out of the scope of this article: some important studies, although the list is not in any way exhaustive, are the ones collected in Shiffrin and Börner (2004), Scharnhorst et al. (2012), and Batagelj et al. (2014).

Despite these attempts, the mapping of what we can call multi-mode networks (Fararo and Doreian 1984; Carley 2003) remains a highly complex task that can be difficult to achieve: the majority of studies thus concentrate on one type of node or of relation, mainly on co-authorship and on citations. However a complementary stream of research has emerged that does not only look at the outputs of research, but untangle the architecture of collaborations during the unfolding process of scientific work by looking at collaborations to research projects. Such studies have mainly looked at European framework programmes and measured the structure of collaborations between the public and the private sector, its evolution over time and its relation to regional and national innovation processes and economic development (Breschi and Cusmano 2004; Protogerou et al. 2010; Katz 1994; Maggioni and Uberti 2007; Scherngell and Barber 2011; Scherngell and Lata 2013; Maggioni et al. 2014).

What these studies clearly show, as Mali and colleagues summarise, is that “science never operates as a single community with hundreds of thousands of individual scientists. It is organized by many different networks that cut across the formal boundaries dividing science with regard to disciplinary, sectoral, and geographical levels. Of course, the membership of various networks overlaps considerably. These research networks are also in continuous processes of growth, decline, and dissolution” (Mali et al. 2012: 201). Studies have shown, for example, how European funding policies, and specifically framework programmes (FP), have promoted increasing cohesion of European research, and how cohesion is sustained by an oligarchic core of scientists that concentrate research collaborations around them, sometimes at the expenses of peripheral institutions and research groups (Breschi and Cusmano 2004). Other studies have investigated the impact of geographical distance (Katz 1994; Maggioni and Uberti 2007; Scherngell and Barber 2011; Scherngell and Lata 2013), of language barriers (Scherngell and Barber 2011; Scherngell and Lata 2013) and of sector similarities (Maggioni and Uberti 2007; Scherngell and Barber 2011) in establishing collaborations.

A fruitful debate has thus concentrated on collaborations across disciplines. Gibbons et al. (1994), for example, distinguish between ‘mode 1’ interdisciplinary research, where multiple disciplinary approaches help to advance a speciality, or to move it toward new applicative areas; and ‘mode 2’ interdisciplinary research, more problem oriented and project specific. This distinction between theory driven and application driven research is expanded in Bruce et al. (2004), where they distinguish between ‘multidisciplinary’ research, where various disciplines are juxtaposed with little cross-fertilization; ‘interdisciplinary’ research, where different disciplinary contributions are integrated in a systemic outcome; and ‘transdisciplinary’ research, tailored to specific problems and open to collaborations external to Academia. Various authors have also reflected upon the relationship between disciplinary and interdisciplinary research, as efficiently summarised in Jacobs and Frickel (2009: 54–57). Abbott (2001) sees interdisciplinary research as a residual system of disciplinary research, where the former is problem oriented and thus not in competition with the theory laden research of disciplines. Turner instead (2000) see disciplinary and interdisciplinary research as independent systems, where the latter creates new areas of enquiry. For Whitley (1984) interdisciplinary research is not simply a residual outcome of disciplinary research, but the two fields are growing increasingly interdependently and the relationship is mutually influencing. Finally, Fuller (1991) sees disciplinary research as conventional rhetoric constructs that are solidified by dominant social circles and grant access to funding resources. In his words, “disciplinary boundaries provide the structure needed for a variety of functions, ranging from the allocation of cognitive authority and material resources to the establishment of reliable access to some extra-social reality” (Fuller 1991:302). As boundaries are socially established, they can be constructed, maintained and deconstructed: what these boundaries mark is “the point at which methods are institutionalized, or, so to speak, the word is made flesh” (Fuller 1991: 302). Because disciplinary boundaries tend to radicalise around “canonical histories” and specific “cults of expertise” (Fuller 1991), the real source of epistemic change is to be found in interdisciplinary research (Fuller 2003), which cut across such boundaries and refuse to conform to orthodox disciplinary research.

The role of disciplines in establishing demarcation criteria thus does not only serve the purpose of systematizing the ontological and epistemological domains of science, but more important enable scientists to access political and material resources, including the seats of power themselves (Abbott 2001). This is clearly reflected by the disciplinary organization of Italian Academia, which is ruled by the Ministry of Research and University (MIUR) and has implications for the system of academic recruitment and research funding. In the next section we briefly illustrate the organization and progressive systematization of Italian academic Scientific-Disciplinary Sectors (SSD) which are the constitutive ground of our research: the review shows how far from being stable and watertight containers, disciplines’ boundaries fluctuate over time to accommodate the social and political organization of Academia.

## MIUR and SSD: the organization of Italian Academia and the system of PRIN funding

The Ministry of University and Research (MIUR) organises Italian Universities. MIUR is in charge of recruiting, evaluating and assigning academic staff (researchers, lecturers, professors) to disciplines, of defining the overall content of academic degrees, and of distributing research funding. These activities are regulated according to the scientific-disciplinary sectors (SSD) of research and teaching, which were first defined in 1973 in order to organise the national recruiting system. SSD grew from 263 to 430 within the space of 10 years (1973–1983), were further modified in 1994 and in 1999 were reduced to 370 and aggregated in 14 macro-disciplinary areas. The last re-organization of disciplines was done in 2008, when recruitment sectors (191) and macro-sectors (86) were introduced, merging together several SSD in order to facilitate the recruitment process.^1^ Areas represent whole disciplines (e.g.: Physics), while SSD define specific sub-disciplinary sectors (e.g.: Theoretical Physics, Material Physics, Astrophysics, and the like). Academic staff is recruited on the grounds of disciplinary expertise: every scientist can only be appointed in Academia after being evaluated by a public scientific commission, organised by MIUR, which reviews his/her curriculum vitae and judges not only the quality and quantity of scientific outputs, but also their relevance for the SSD the candidate applies for. If the evaluation process is successful the scientist is assigned to a disciplinary area and its relevant SSD. When a scientist applies for a promotion (for example, to upgrade from the role of researcher to the one of associate professor, and from the role of associate to the one of full professor), a new evaluation takes place that revises the consistency of scientific outcomes for the SSD s/he belongs to, which may confirm the scientist’s affiliation or may require a change of SSD. The MIUR website provides updated individual SSD affiliation for every scientist appointed in Academia: although they could potentially change disciplinary affiliation during their career, this only happened for 0.8 % of the scientists funded in PRIN projects in the 10 years under analysis.

Currently disciplinary areas are categorised as in Table 1, which also aggregates them in the traditional division between Natural and Technical Sciences and Humanities and Social Sciences, and compares these areas with the corresponding European Research Council (ERC) panels:

**Table 1:** Categorization of disciplinary area and number of sub-disciplines in each area according to MIUR, and the equivalent ERC panels

Area code	Disciplinary area	N. sub-disciplines	ERC panels’ categorization
Natural and Technical Sciences			
1	Mathematics and information science	10	PE1; PE6
2	Physics	8	PE2; PE9
3	Chemistry	12	PE4; PE5
4	Environmental sciences	12	PE10
5	Biology	19	LS1; LS2; LS3
6	Medicine	50	LS4; LS5; LS6; LS7
7	Agriculture and veterinary	30	LS8; LS9
8	Architecture and civil engineer	22	PE8
9	Industrial and information engineer	42	PE3; PE7
Humanities and Social Sciences			
10	Ancient studies, literature and philology, history and art	77	SH5; SH6
11	History, philosophy, pedagogy and psychology sciences	34	SH4; SH6
12	Law studies	21	SH2
13	Economics and statistics	19	SH1
14	Political and social sciences	14	SH2; SH3

This brief overview of the organization and historical evolution of disciplines in Italian Academia, efficiently summarised by Pascuzzi (2012), shows that disciplinary areas and their SSD, far from being stable and universal classifications of science, are subjected to progressive modifications. By regulating the access to academic career, they play a strategic role in the distribution of public funding to Universities. Disciplinary areas and SSD also define the content of academic degrees, which is likewise intertwined with the system of recruitment: universities must teach specific sub-disciplines in order to be able to grant degrees, and therefore must appoint staff accordingly. The various reconfigurations of SSD do not only satisfy the need of updating scientific knowledge classifications, but they are also useful to increase or decrease the number of academic staff that need to be appointed, and to re-organise the access to academic jobs.

More important for the scope of this article, MIUR is in charge of distributing research funding: again, funding are organised around disciplinary areas, although they do not impede interdisciplinary projects. In particular, MIUR allocate a yearly budget to fund what are called Research Projects of National Interests (PRIN) which for some disciplines, especially Humanities, represent the main source of research funding. The PRIN scheme is a form of co-funding between MIUR and universities: every year, researchers obtain a budget from their universities to cover 30 % of a research project’s cost, with the other 70 % provided by MIUR if the project is positively evaluated and subsequently funded. Projects are organised as inter-organizational collaborations between researchers based in different universities, where each project is led by a national coordinator (principal investigator), and involves various local units. These are normally based in different institutions, although there can be exceptions in which coordinators of local units of the same project may belong to the same department. The PRIN scheme does not include any funding priorities, but are open calls in which any topic in any research field can be investigated, and where projects are evaluated according to the quality of the PI and of the Units’ coordinators profiles, the originality, methodological adequacy, impact and feasibility of the project, and the availability of funding from the applicant’s institutions.

Information on funded projects is available from the MIUR website (www.miur.it). Every funded project for every year (since 1996, although our analysis starts from 2001) is listed in a pdf file containing the name and University affiliation of researchers together with their role (national coordinator, local unit coordinator), the amount of funded money for each unit, and the title of the project. This is followed by a general account of the aim of the research, a statement about innovations in the topic of enquiry, a list of criteria for the verifiability of the project’s outcomes, and finally a detailed description of each research team’s duties. MIUR website also provides information about scientists’ disciplinary affiliation (SSD). The data analysed in this paper cover the period between 2001 and 2010 which we aggregated in three time slots (2001–2004; 2005–2007; and 2008–2010) to allow and facilitate longitudinal analysis.

As we said before, each discipline in Italian Academia is internally subdivided into sub-disciplinary sectors (SDD) which distinguish, for example, between theoretical and experimental physics, or between ancient and modern philosophy. The topic of a PRIN project must be related to the disciplinary area in which it is proposed and eventually funded; however units’ coordinators can be affiliated to various sub-disciplinary sectors, or even to different disciplinary areas. Thus, for example, it is possible to have a project (Table 4) where rural economists (AGR/01) collaborate with horticulturists and floriculturists (AGR/04) (collaboration within the same disciplinary area although not in the same SSD sector), or where rural economists (AGR/01) collaborate with environmental botanists (BIO/03) (collaboration between different disciplinary areas).

**Fig. 1:**
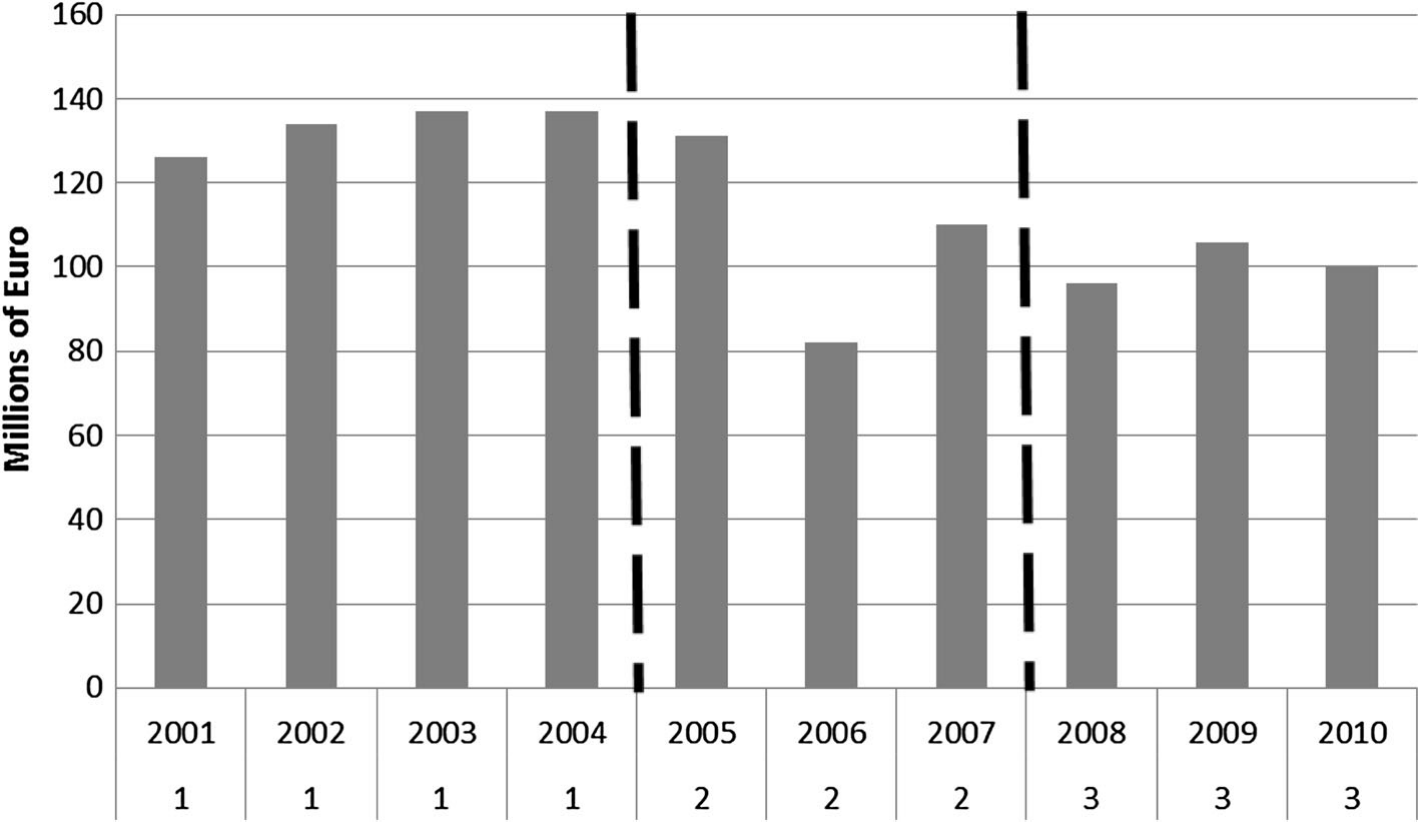
Total amount of money funded for PRIN projects (millions of euro). *Numbers* on *X* axis indicate the three time slots in which data have been divided, also highlighted by the dotted lines

MIUR website reports aggregate data on the amount of money allocated through the years for PRIN projects, and on their rate of success. Figure 1 reports the total amount of money allocated from 2001 to 2010: while there haven’t been any notable change between 2001 and 2005 (around 130 million of euro per year), in 2006 funding were drastically reduced (82 million). In the following 4 years there was an increase (around 100 million), but not enough to bring the amount of funding back to the pre-2006 levels.

Likewise, from our data on funded projects we calculate the number of funded scientists, funded projects, average number of scientists per projects, and total amount of money funded per disciplinary area for the decade under analysis, summarised in Table 2. While overall some disciplines receive more funding (although this does not indicate a better rate of success, as other disciplines may receive funding from other sources) and have a higher number of projects and people funded, the average size of research groups does not vary dramatically across disciplines, ranging from 3.57 of biology to 5.11 of architecture and civil engineering, but mostly assessing around 4 members. Also, the average size of research group does not correlate with the average amount of money funded (Pearson Correlation −0.17, 2tail sig 0.545), indicating that funding are not allocated simply by looking at how many people participate to a project.

**Table 2:** Number of funded scientists, funded projects, average number of scientists per projects, and total amount of money funded per disciplinary area

Area code	Disciplinary area	N. of funded scientists	N. of funded projects	Average n. of people per project	Total amount of funded money (€)
Natural and Technical Sciences					
1	Mathematics and information science	653	315	4.69	43,429,650
2	Physics	957	442	3.82	90,264,515
3	Chemistry	1198	475	4.89	134,459,919
4	Environmental sciences	590	288	3.71	35,299,403
5	Biology	1991	1053	3.57	177,747,765
6	Medicine	3411	1391	4.21	205,540,380
7	Agriculture and veterinary	1542	636	4.14	67,781,287
8	Architecture and civil engineer	1324	420	5.11	73,722,258
9	Industrial and information engineer	1994	779	4.26	128,066,247
Humanities and Social Sciences					
10	Ancient studies, literature and philology, history and art	1509	655	4.27	69,468,903
11	History, philosophy, pedagogy and psychology sciences	1311	497	4.57	59,455,777
12	Law studies	1219	509	4.54	36,775,681
13	Economics and statistics	1067	422	4.10	37,206,371
14	Political and social sciences	676	283	4.31	33,681,076

Years 2001–2010

**Fig. 2:**
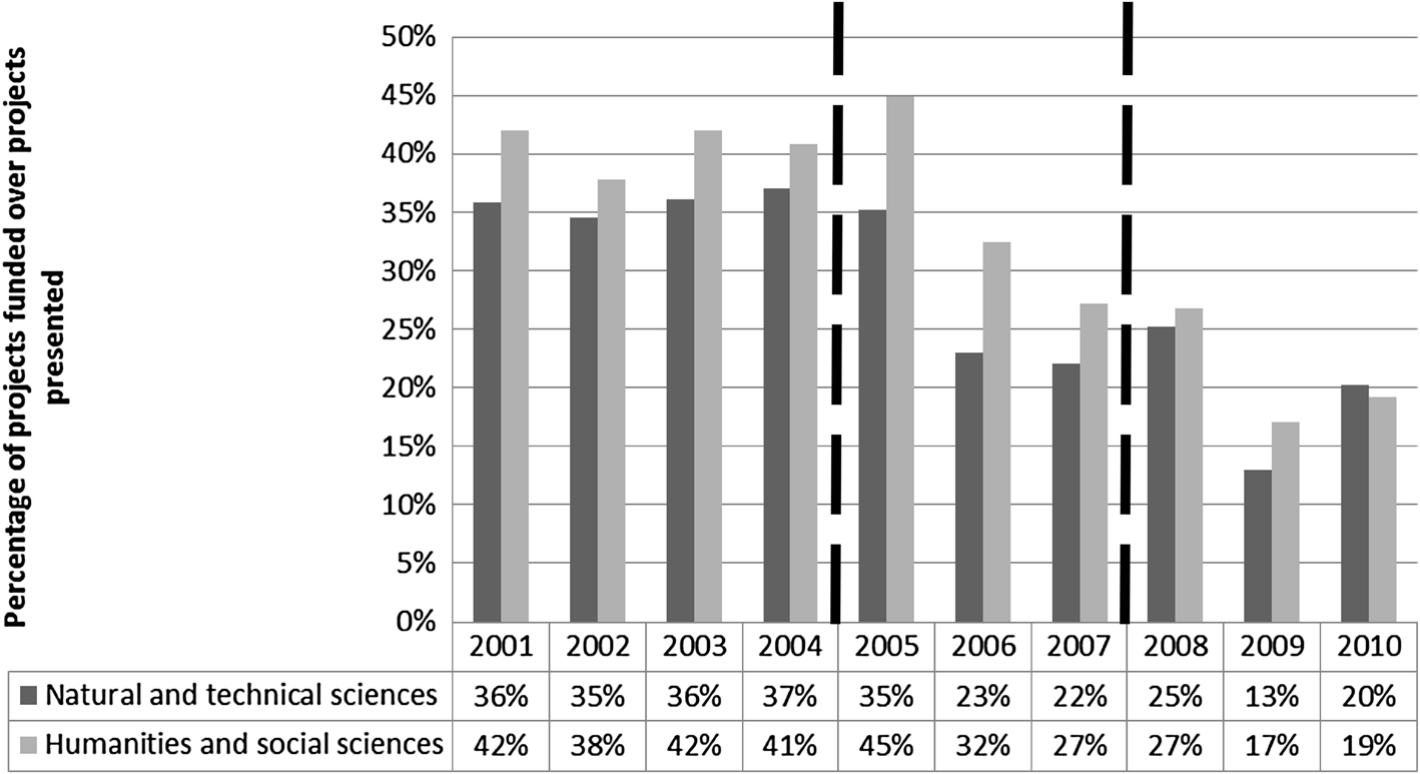
Percentage of projects funded over projects presented each year in each discipline, where disciplines are aggregated in Natural and Technical Sciences versus Humanities and Social Sciences. The two values do not add up to 100 %, as they indicate the rate of success in each macro area respectively

The funding reduction seemed to have a direct impact on the rate of success of PRIN projects, measured by the percentage of projects that received funding over the total number of projects presented in each disciplinary area. Figure 2 represents this measure, and aggregates the percentage of successful projects in two macro-areas: Natural and Technical Sciences, and Humanities and Social Science. It also shows how such percentage seems to follow a similar trend of the funding allocation, remaining more or less stable between 2001 and 2005, to then dropping from 2006 to 2009, with two minor increases in 2008 and 2010. The same figure indicates the better rate of success of Humanities and Social Sciences projects over the natural science ones, although in 2010 the gap was filled and eventually reversed.^2^

**Fig. 3:**
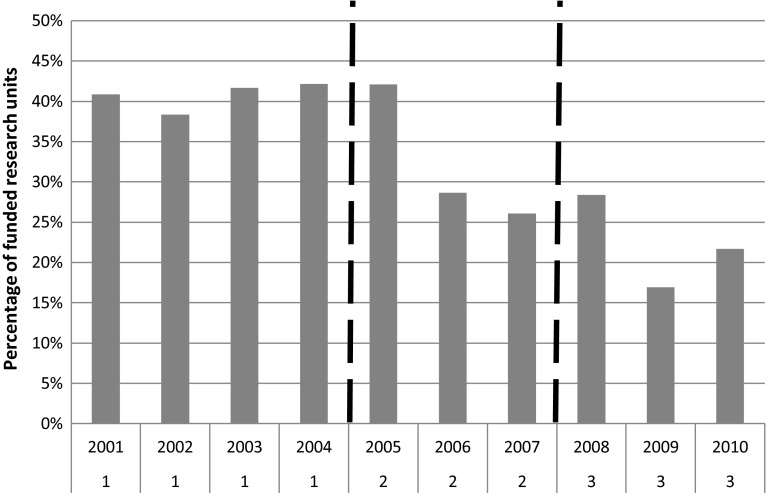
Percentage of funded research units over presented. *Numbers* on *X* axis indicate the three time slots in which data have been divided, also highlighted by the *dotted lines*

A similar trend is noticeable in the percentage of research units funded (Fig. 3), which drastically decreases from 2006, with only to minor increases in 2008 and 2010.

The funding reduction is also correlated to the average size of research groups (Pearson Correlation 0.78, Sig 2 tailed 0.008): when funding is reduced, the average size of research groups also diminishes.

Finally, the reduction of funding also seems to relate to an increasing cohesion of the collaboration network. Table 3 summarises the standard cohesion measures of the networks of collaborations between SSD in the three time periods. While the first and third period under analysis present similar characteristics the middle period, which coincides with the funding decrease, departs from the other two. The average degree and the density of the whole network increase, as well as the size of the main component; while conversely the number of components (and consequently the fragmentation), the average distance and the diameter decrease. These tendencies combined indicate that during the middle period the cohesion of the network increases.

**Table 3:** Descriptive statistics of the SSD by SSD network for the three time slots

	2001–2004	2005–2007	2008–2010
Total no. SSD	370	370	370
Avg degree	4.81	6.08	4.74
Density	0.01	0.02	0.01
Size of main component	270	302	271
Components	76	47	50
Component ratio	0.21	0.13	0.15
Fragmentation	0.41	0.26	0.33
Avg. distance	4.11	3.83	4.23
SD distance	1.42	1.28	1.49
Diameter	10	9	10

Avg degree: the average degree in the underlying graph. Density: number of edges divided by the maximum number possible. Components: number of components. Component ratio: number of components minus one divided by the number of actors minus one. Fragmentation: proportion of pairs of vertices that are unreachable. Avg. distance: average geodesic distance amongst reachable pairs. SD distance: standard deviation of the geodesic distances amongst reachable pairs. Diameter: length of the longest geodesic (Borgatti et al. 2002)

The reduction of funding for PRIN projects poses some noteworthy questions in regards to research collaborations. An interesting task would be to investigate if the increased cohesion of the network, which seems to correspond to a concentration of resources on a smaller number of research groups, indicate the existence of an oligarchy structure as already observed for the European context (Breschi and Cusmano 2004). We know that the number of funded research units and average research group size reduces together with funding reduction, but from our data we cannot see if this also implies a concentration of resources on few successfully recurrent group leaders. Further analysis is required in this direction which falls beyond the scope of this article.

What we want to do here is to concentrate on the aspect of disciplinary and interdisciplinary research. It is without doubt that in recent years there has been a growing call for interdisciplinary research (Hicks and Katz 1996; Katz and Martin 1997; Cummings and Kiesler 2005; Jacobs and Frickel 2009), as testified for example by the clear call for integrated research approaches in the European Commission’s Framework Programmes (Bruce et al. 2004; Breschi and Cusmano 2004; Maggioni et al. 2014). We thus want to analyse the longitudinal evolution of scientific research in Italian Academia by looking at disciplinary and interdisciplinary collaboration, to see if such calls have been followed by Italian scientists. In particular, we want to address the following research questions:

- How much interdisciplinary research has been conducted in Italy in the decade 2001–2010 under the PRIN scheme?
- Looking at funded projects, has the number of interdisciplinary collaboration increased or decreased in the 10 years under analysis?
- Are there any differences between disciplinary areas? Can we find common patterns of disciplinary and interdisciplinary research across disciplinary areas? Do they reflect the traditional division between natural sciences versus humanities and social sciences?
- Do these patterns change over time?
- Looking at funded projects, do the ones with an interdisciplinary component receive an increasing share of funding over time?

In the next section we detail the process of data collection and the analytical strategy we employed to measure disciplinary and interdisciplinary research, and to relate it to the amount of money funded projects receive.

## Data manipulation and analytical strategy

The original dataset we collected consists in 10 bipartite^3^ networks ‘people by funded projects’, one for each year from 2001 to 2010, representing the collaboration of each researcher to a specific project. Overall 19,453^4^ researchers have been funded in 8182 projects. Using scientists’ SSD affiliation we construct a second matrix ‘sub-discipline by people’, and we multiply this second matrix by the first ‘people by funded projects’ matrix, obtaining one ‘sub-discipline by project’ matrix for each year under analysis, for a total of 10 matrices. These derived matrices were transformed into unipartite matrices ‘sub-disciplines by sub-disciplines’ with number of projects in common (Table 4; each matrix consists of 370 × 370 nodes, representing all SSD). Finally, these 10 matrices were summed up in three time slices: 2001–2004/2005–2007/2008–2010, to compare research collaboration across time.

**Table 4:** Extract of a matrix of sub-discipline by sub-discipline with number of projects in common (first 5 rows only: the whole matrix has 370 × 370 nodes, corresponding to all the sub-disciplines)

	AGR/01	AGR/02	AGR/03	AGR/04	AGR/05	BIO/01	BIO/02	BIO/03	…
AGR/01	***31***	**0**	**0**	**1**	**0**	*0*	*0*	*2*	
AGR/02		***102***	**0**	**2**	**0**	*2*	*1*	*0*	
AGR/03			***48***	**0**	**0**	*7*	*0*	*0*	
AGR/04				***47***	**0**	*0*	*0*	*2*	
AGR/05					***48***	*0*	*0*	*0*	
…									

Fonts distinguish between collaborations within the same sub-discipline (bold italic); collaborations outside sub-discipline but within disciplinary area (bold); collaborations outside disciplinary area (italic)

The literature offers two ways for analysing the unipartite matrices obtained from bipartite networks: in some cases only the relationship between the national coordinator and the local units is considered, disregarding the ones between local units themselves; in other cases the full network of relationships between local units, and between these and the national coordinator, is considered (Katz 1994; Scherngell and Lata 2013). In the first scenario it is assumed that the national coordinator acts as an intermediary between the local units, while in the second one all units are in contact with each other, and presumably exchange information (Protogerou et al. 2010).

Scholars have compared the two methods against each other to observe variations in results (Breschi and Cusmano 2004; Maggioni and Uberti 2007; Maggioni et al. 2014; Protogerou et al. 2010), and the general conclusion is that both the assumption are rather strong and somehow arbitrary (Breschi and Cusmano 2004; Protogerou et al. 2010). In our case, without more detailed data we cannot safely assume that the local units do not establish contact with each other and would not further collaborate independently from the national coordinator. Indeed, in a more detailed study of the areas of physics and philosophy, we observed that several members of local units can jointly migrate from one national coordinator to another one over time, becoming brokers between different research groups (Bellotti 2015), potentially indicating a peer referral system in place. One analytical strategy to untangle the dynamics of peer referral processes between research groups could be the one used by Koskinen and Edling (2012) to model the evolution of bipartite networks, although it is not currently applicable to our dataset given the heterogeneity of actors across years. Following Katz (1994) and Scherngell and Lata (2013) we therefore decided to adopt the full network method.

In order to answer our first and second research questions and see how much interdisciplinary research has been effectively conducted in Italy and if interdisciplinary collaboration increase or decrease during the 10 years under analysis, we count, for each of the three matrices:

1. The diagonal values, representing the number of projects in common between each sub-disciplinary sector (SSD). This value represents what we called in-subdiscipline collaboration, e.g.: rural economy (AGR/01) collaborating with rural economy (bold italic cells in Table 4).
2. The off diagonal values, representing:
  - Collaboration outside sub-disciplinary sectors but within the disciplinary area. This value represents what we called in-discipline collaborations, e.g.: rural economy (AGR/01) collaborating with horticulture and floriculture (AGR/04) (bold cells in Table 4).
  - Collaboration outside disciplinary area. This value represents what we called out-discipline collaborations, e.g.: rural economy (AGR/01) collaborating with environmental botanic (BIO/03) (italic cells in Table 4).

We then aggregate the SSD into the 14 disciplinary areas, and consider the proportion of collaboration within these three categories (in-subdiscipline, in-discipline, out-discipline) over the total number of collaboration for each disciplinary area in each time slice. We normalise the number of projects in each category against the total number of projects to make possible a comparison across different disciplines, which have a different overall number of funded projects within the 10 years, ranging from the minimum of 283 projects funded in Social and political sciences (area 14) to a maximum of 1391 projects funded in Medicine (area 6) (Table 2).

For the analysis the data is organised as modal multivalued symbolic data in which each of the three time slices is treated as a symbolic variable (Billard and Diday 2006). In such variables, technically addressed as symbolic objects, the units of analysis are disciplines each represented by the discrete distribution of collaboration in the previously described three categories (in-subdiscipline, in-discipline, out-discipline).

In order to answer our third research questions and see if we can find common patterns of disciplinary and interdisciplinary research across disciplinary areas, and if these patterns change over time, we cluster the disciplines according to the similarity of the distributions. A clustering procedure for the specific type of symbolic objects used in the analysis has been developed by Batagelj et al. (2016) and applied on various datasets e.g. clustering of scientific disciplines according to the distribution of co-authorship (Kronegger et al. 2015), clustering distributions of patent citations (Kejžar et al. 2011) and population pyramids (Korenjak-Cerne et al. 2015). Applied clustering procedures for symbolic data have been implemented in R (R Development Core Team 2012) within the package Clamix (Batagelj and Kejžar 2011).

Like most clustering algorithms the used one is based on distance or (dis)similarity measure between pairs of units. The definition of the similarity measure is the most important transition from the traditional clustering approaches to the clustering of symbolic data. If each analysed variable *V*_*i*_ (*i* = 1, …, *M*) is described by a distribution (vector) *x*_*i*_ of its values, we assume that *T* is a representative of the cluster *C* and is also described by *M* distributions. The dissimilarity between a unit *X* from the cluster *C* and its representative *T* equals

$${d\left( {X,T} \right) = \sum {\alpha_{i} } \times d\left( {x_{i} ,t_{i} } \right),\alpha_{i} \ge 0,\sum {\alpha_{i} } = 1}$$

where *x*_*i*_ and *t*_*i*_ are relative distributions of the unit *X* and the representative *T* respectively, for the variable *V*_*i*_ with *k*_*i*_ categories, and

$$d\left( {x_{i} ,t_{i} } \right) = w_{xi} \times x_{i} - t_{i}^{2} = w_{xi} \sum {\left( {x_{ij} - t_{ij} } \right)^{2} }$$

where *w*_*xi*_ > 0 is a weight of variable *V*_*i*_ for the unit *X*. A value *α*_*i*_ is used to tune the importance of each variable *V*_*i*_.

A hierarchical clustering procedure starts with each unit as a separate cluster and proceeds with step-by-step merging of the two closest clusters. After each fusion, the distances between the new (fused) cluster and the remaining clusters are determined. In our application the Ward’s clustering method is used (Ward 1963). Clustering of symbolic data requires the generalised version of Wards clustering mechanism provided by Korenjak-Černe et al. (2011).

Initially we run hierarchical clustering routine on three time slices together (as separate symbolic variables) to obtain single clustering solution and answer the first part of our third research question (3a). Then we run the same routine for each of the three time slots (2001–2004/2005–2007/2008–2010) separately to answer the second part of our third research question (3b). The clusters resulting from the single solution analysis represent disciplines with similar distributions of collaboration across the three categories (in-subdiscipline, in-discipline, out-discipline): by comparing the clusters over the three time slots we then check the stability of clustering and the movements of disciplines between clusters over time.

Finally, to address our fourth research question and see if projects with an interdisciplinary component receive an increasing share of funding over time, we measure the amount of money PRIN projects receive in each discipline in each time period, and we then correlate it with the number of collaborations in-subdiscipline, in-discipline, and out-discipline within the same period.

Our data present some limitations in terms of what they can effectively represent. First of all, they only cover a specific line of funding for Italian Academia. There are other sources of research funding in Italy, especially for disciplines like Medicine, Biology and Physics that can access vast amount of private funding. However PRIN projects are the main line of public funding to Italian research, therefore it is interesting to analyse them. Second, we do not have information on unsuccessful bids, as MIUR website only publish projects that have been successfully funded. This, combined with the lack of information on different source of funding, means that if researchers or sub-disciplines haven’t been funded via PRIN projects in the years under analysis they are not necessarily unsuccessful. It could be that they have been funded via other sources, or that they have not participated to the funding competition at all. Third, data only refer to Italian Academia, and therefore results may vary significantly in other countries. Fourth, data only refer to the decade 2001–2010. It would have been interesting to compare previous years, but the MIUR database does not provide exhaustive information on previous years. Fifth and final, although we are interested in looking at the correlation between the percentage of collaborations within and across disciplines and the amount of funding disciplines receive over time, we cannot establish a causal link between the two, as it could be that an increase of funding causes a larger investment in multi-disciplinary research, as well as multi-disciplinary research attracts more funding. Therefore we limit our analysis to correlations, avoiding the temptation to regress one phenomenon against the other. Also, research budgets include different expenses, like staff time allocation, equipment, travelling costs, etc., which vary across discipline and are usually research specific. We do not have access to the breakdown of projects’ budgets, therefore we cannot control for the different distribution of funding.

## Results: distribution, longitudinal patterns and funding of disciplinary and interdisciplinary collaborations

**Fig. 4:**
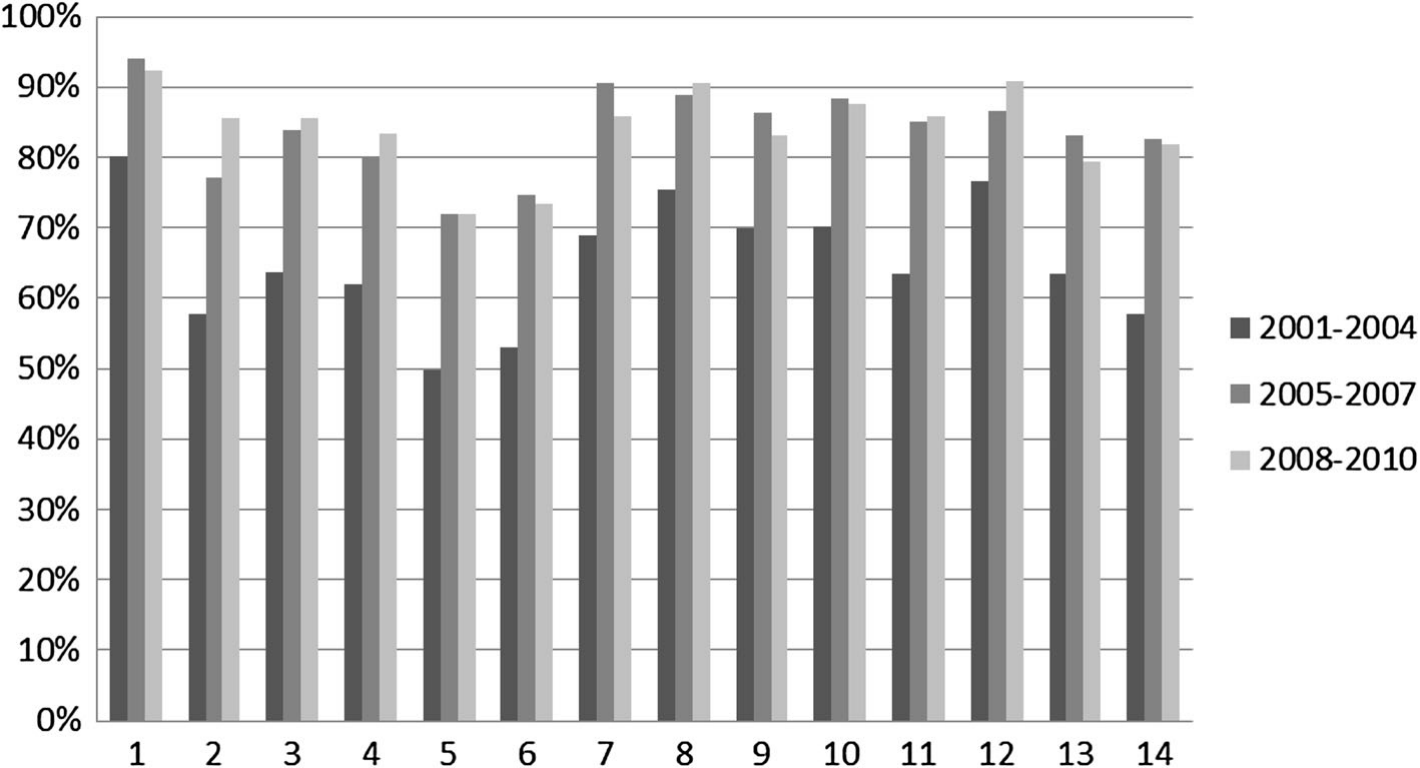
Percentage of collaborations in-subdisciplines

**Fig. 5:**
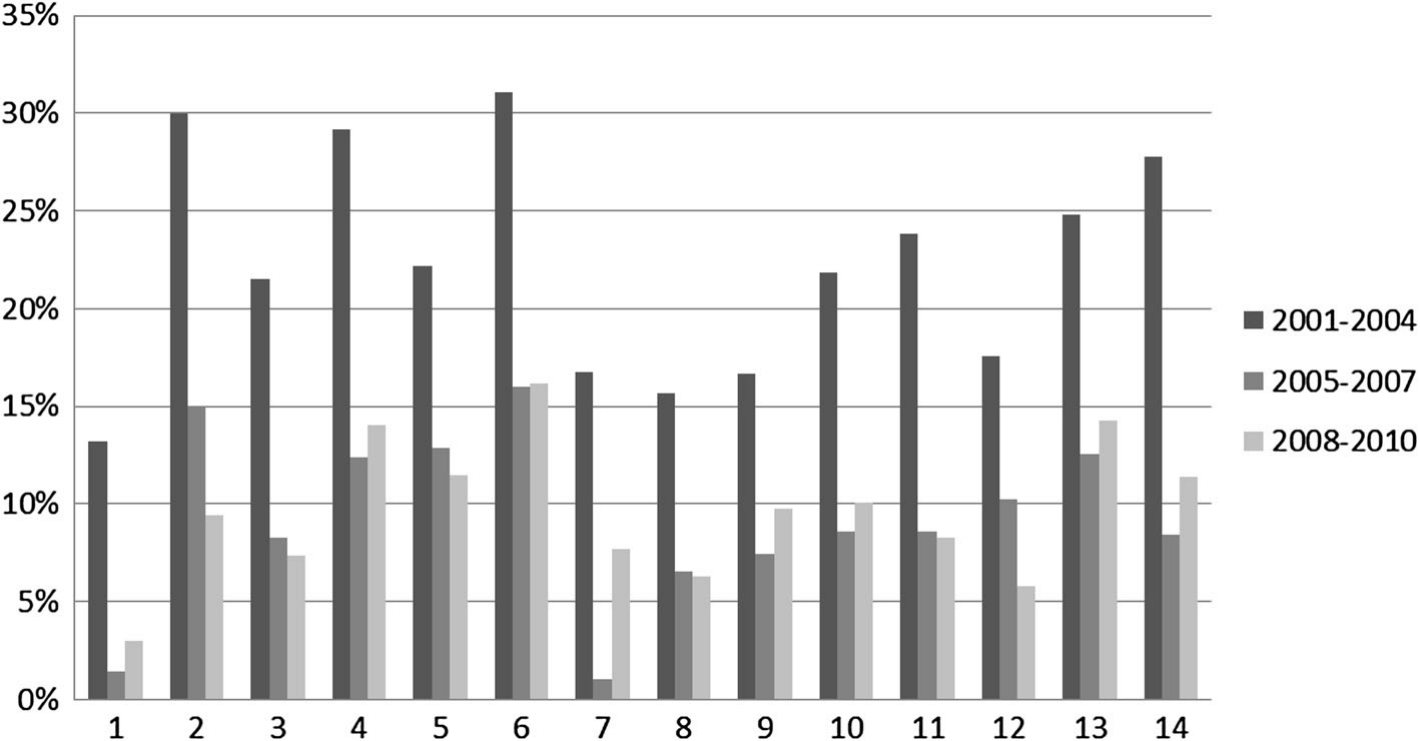
Percentage of collaborations in-discipline

**Fig. 6:**
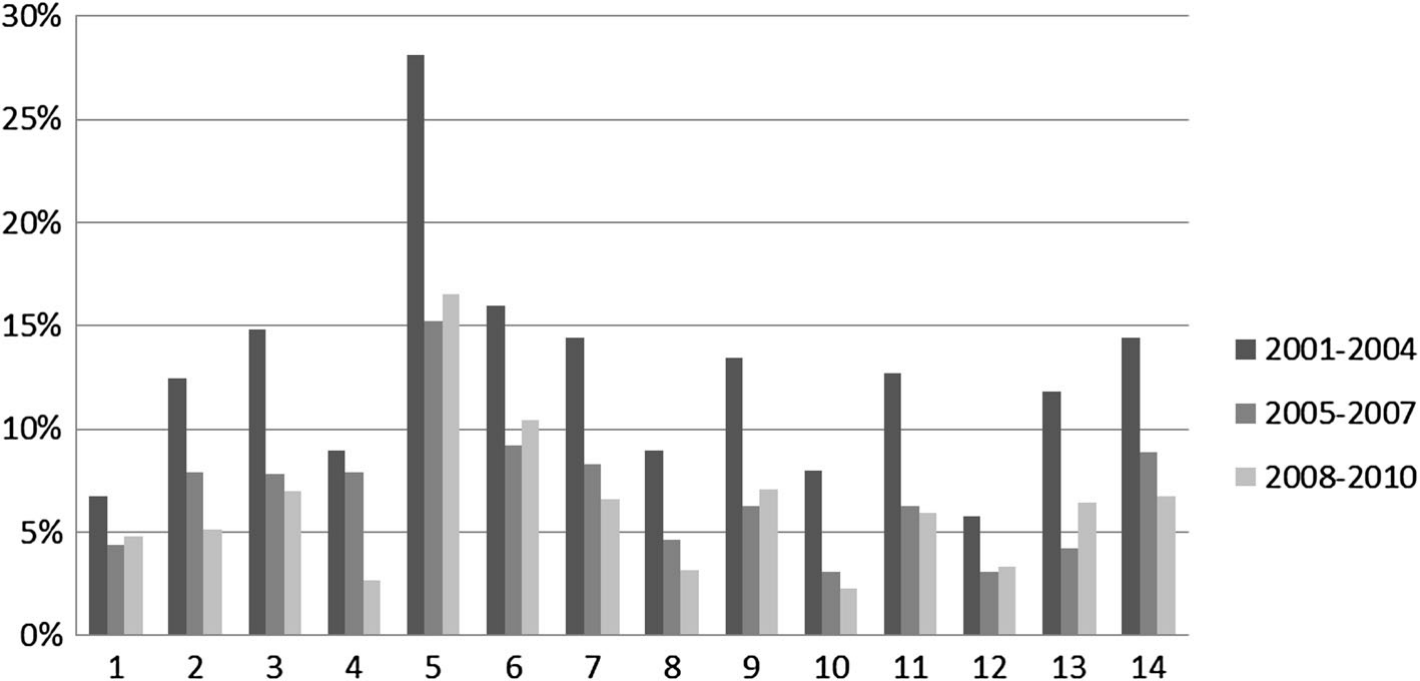
Percentage of collaborations out-disciplines

**Fig. 7:**
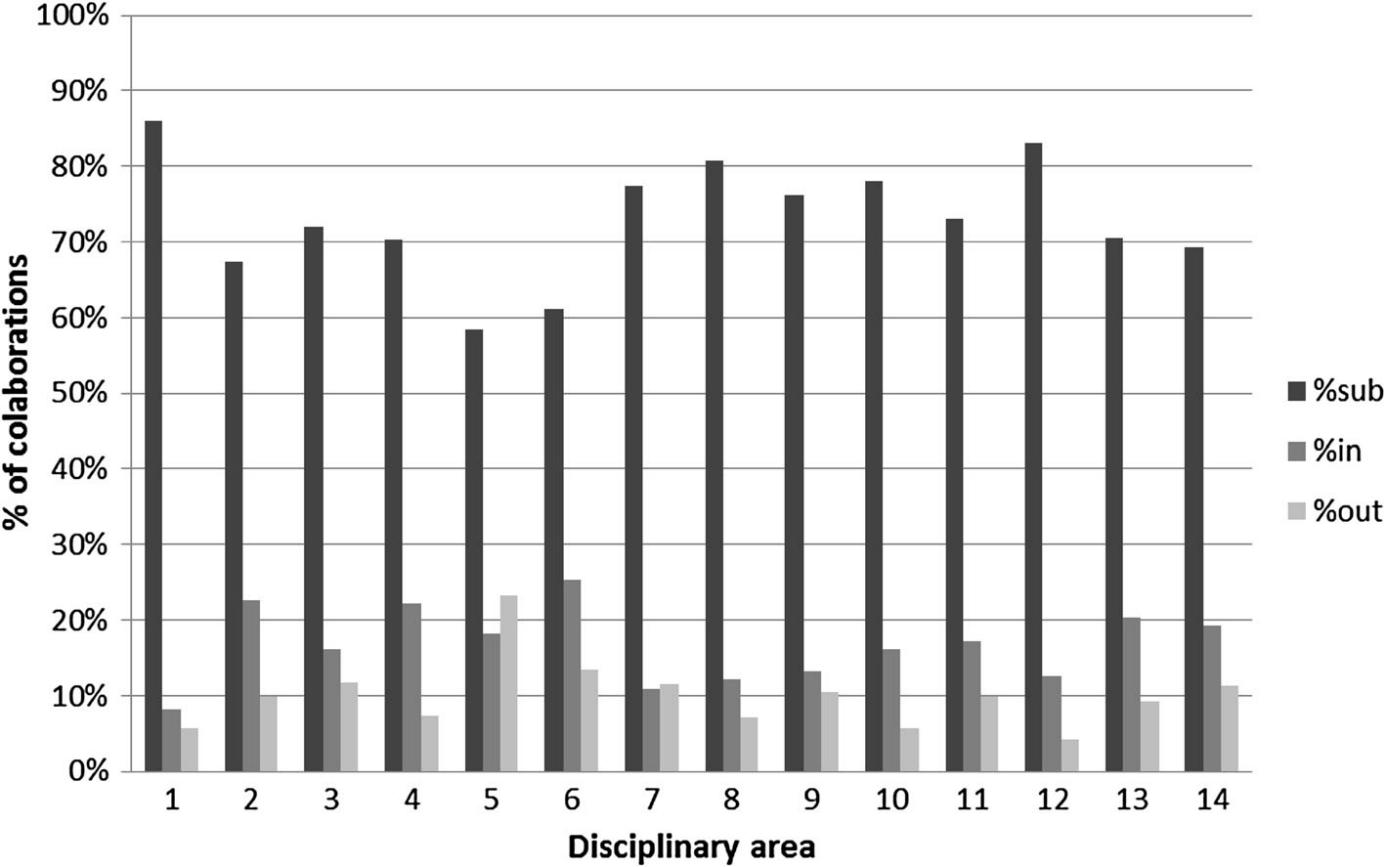
Overall proportion of sub-discipline, in-discipline and out-discipline collaborations per disciplinary area

We now move to the first part of the analysis that looks at the distribution of in-subdiscipline, in-discipline, out-discipline collaboration over the total number of collaborations for each disciplinary area in each time slice. Figures 4, 5 and 6 respectively illustrate the percentage of in-subdiscipline; of in-discipline; and of out-discipline ties for each disciplinary area for the periods 2001–2004; 2005–2007; 2008–2010 (visualised in different columns’ colours). Figure 7 summarizes the overall proportion of sub-discipline, in-discipline and out-discipline collaborations per disciplinary area.

From Fig. 4 it is clear that the highest percentage of research collaboration is activated within the same sub-discipline, and it ranges from 50 % of the total number of collaborations in 5. Biology to the 80 % of 1. Mathematics and informatics in 2001–2004. All the disciplines increased this percentage in the years between 2005 and 2007, where the range spans from 72 % for 5. Biology to 94 % of 1. Mathematics and informatics, and the increase continue in 2008–2010, although in the same period nearly half of the disciplines slightly reduced the investment in sub-disciplinary collaboration (1. Mathematics and Informatics; 6. Medicine; 7. Agriculture and Veterinary; 9. Industrial and Information Engineer; 10. Ancient Studies, Literature and Philology, History and Art; 13. Economics and statistics; 14. Political and social sciences). Overall (Fig. 7), the tendency over the years has been toward an increase in sub-disciplinary research, which dominates disciplines like 1. Mathematics and information science; 8. Architecture and civil engineer; and 12. Law studies (>80 %), while is less prominent in disciplines like 2. Physics; 5. Biology; 6. Medicine; and 14. Political and social sciences (<70 %).

Figure 5 shows the percentage of research collaboration activated within the same disciplinary area but outside the same sub-discipline. There can be projects where, for example, theoretical physicists collaborate with material physicists; molecular biologists with pharmacology; urban sociologists with cultural sociologists, and the like. Such collaborations have been dramatically reduced from 2001 to 2004 to the next time slice (2005–2007) across all disciplines. While in 2001–2004 they ranged from 13 % (1. Mathematics and information science) to 31 % (6. Medicine), in 2005–2007 the range dropped to a minimum of 1 % (1. Mathematics and information science and 7. Agriculture and Veterinary) and a maximum of 16 % (6. Medicine). The drop continued in 2008–2010, although in-discipline collaborations rose back for 1. Mathematics and information science; 4. Environmental sciences; 9. Industrial and Information Engineer; 10. Ancient studies, Literature and Philology, History and Art; 13. Economics and statistics; 14. Political and social sciences; and especially for 7. Agriculture and veterinary. Overall (Fig. 7), the tendency over the years has been toward a decrease in in-disciplinary research, which is more popular in disciplines like 2. Physics; 4. Environmental sciences; 6. Medicine; and 13. Economics and statistics (>20 %), and less attractive for disciplines like 1. Mathematics and information science; 7. Agriculture and veterinary; 8. Architecture and civil engineer; and 12. Law studies (<13 %).

Finally, Fig. 6 shows the percentage of collaborative research across disciplinary areas, where, for example, biologists may work with medical scientists; philosophers with political scientists, engineers with chemists. Similarly to in-disciplinary research, collaborations across disciplines represent a minor investment for all disciplines, and they have decreased from 2001–2004 to 2005–2007. In 2001–2004 they ranged from 6 % for 12. Law studies to 28 % for 5. Biology, but the range dropped between 4 % for 1. Mathematics and information science and 13. Economics and statistics; and 15 % for 5. Biology in 2005–2007. In 2008–2010 there has been a minor increase in 1. Mathematics and information science; 5. Biology; 6. Medicine; 9. Industrial and Information Engineer; and 13. Economics and statistics, while the percentage kept dropping for all the other disciplines. Overall (Fig. 7), research across disciplinary areas is more popular for 3. Chemistry; 5. Biology; 6. Medicine; 7. Agriculture and veterinary; and 14. Political and social sciences (>11 %), while is less common in 1. Mathematics and informatics; 10. Ancient studies, Literature and Philology, History and Art; and 12. Law studies (<6 %).

**Fig. 8:**
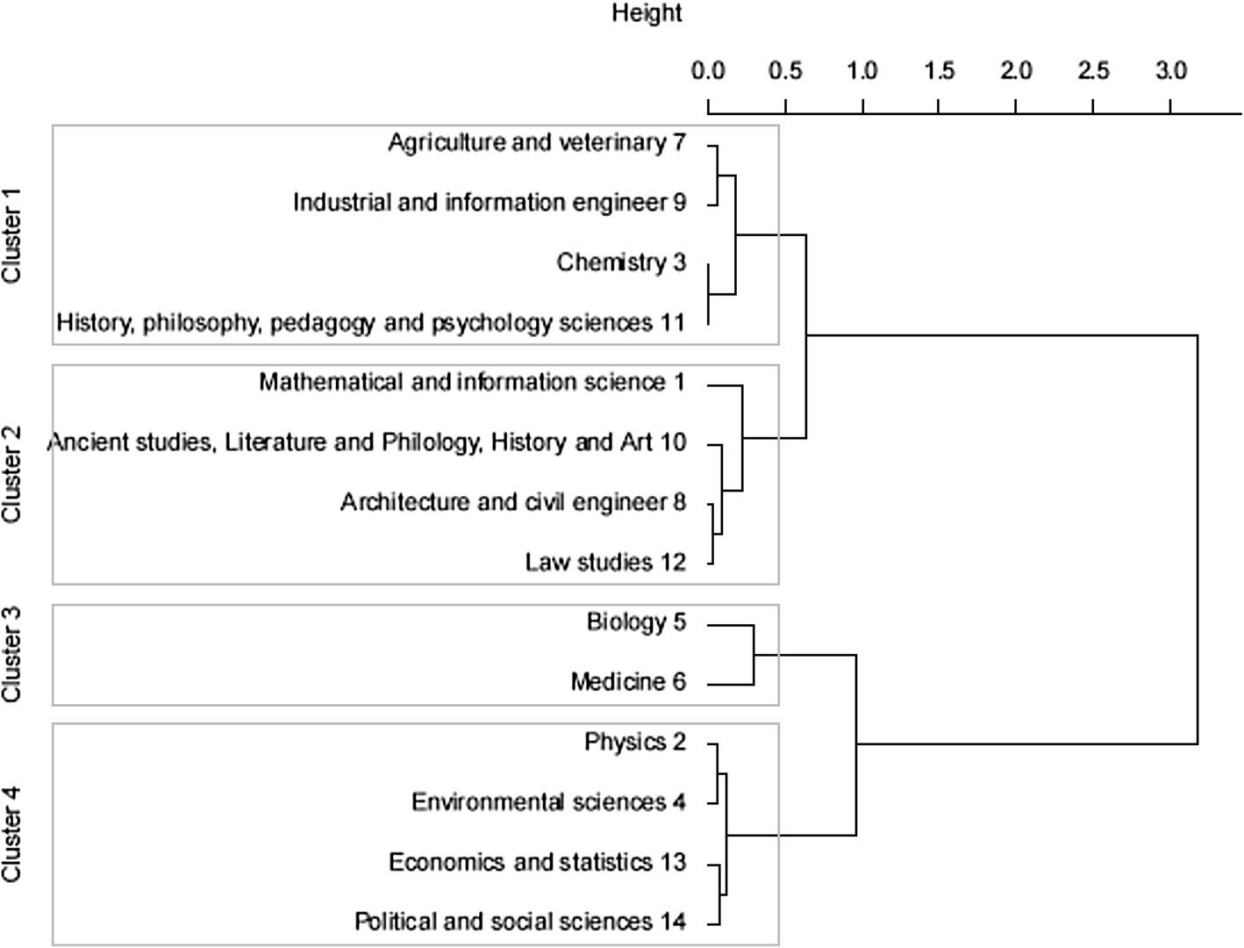
Ward clustering, single solution

The second part of the analysis searches for common patterns of disciplinary and interdisciplinary research across disciplinary areas. We clustered the percentage of in-subdiscipline, in-discipline, and out-discipline collaborations for each disciplinary area firstly without differentiating across the time slices, and results are illustrated in Fig. 8. Here we can see that disciplines merge in 4 clusters (height = 0.5) which include:

Cluster 1: 7. Agriculture and veterinary; 9. Industrial and information engineer; 3. Chemistry; 11. History, philosophy, pedagogy and psychology sciences. This is somehow an odd cluster of difficult explanation. While 3. Chemistry, and 7. Agriculture and veterinary are more inclined toward interdisciplinary research compared to other disciplines (as we have seen in the previous analysis), 9. Industrial engineer and 11. History, philosophy, pedagogy and psychology sciences tend to have a more average profile across the various types of collaborations. They are all, however, experimental and applied areas of research (with the exception of part of 11. History and Philosophy).

Cluster 2: 1. Mathematics and information science; 8. Architecture and civil engineer; 10. Ancient studies, Literature and Philology, History and Art; 12. Law studies. The cluster represents what we can call soft sciences with the inclusion of 1. Mathematics, which already showed behaviour similar to humanities in previous research (although in a different national context, see Kronegger et al. 2015). They are all non-experimental research, based on theoretical modelling (and design) or archival data, and they all focus on sub-disciplinary research.

Cluster 3: 5. Biology and 6. Medicine. This cluster is indicative of the strength of collaborations between these two disciplines, which are also the most incline to interdisciplinary research.

Cluster 4: 2. Physics, 4. Environmental Science, 13. Economics and Statistics, 14. Political and Social Sciences. This cluster merges disciplines that deal with the human and natural environment (natural, economic, political and social). Apart from 2. Physics, which is an odd presence in the cluster (although it could be argued that it similarly deals with the environment), the other disciplines are not grounded on experiments but on empirical observations and data collection. All disciplines are devoted to in-disciplinary research.

**Fig. 9:**
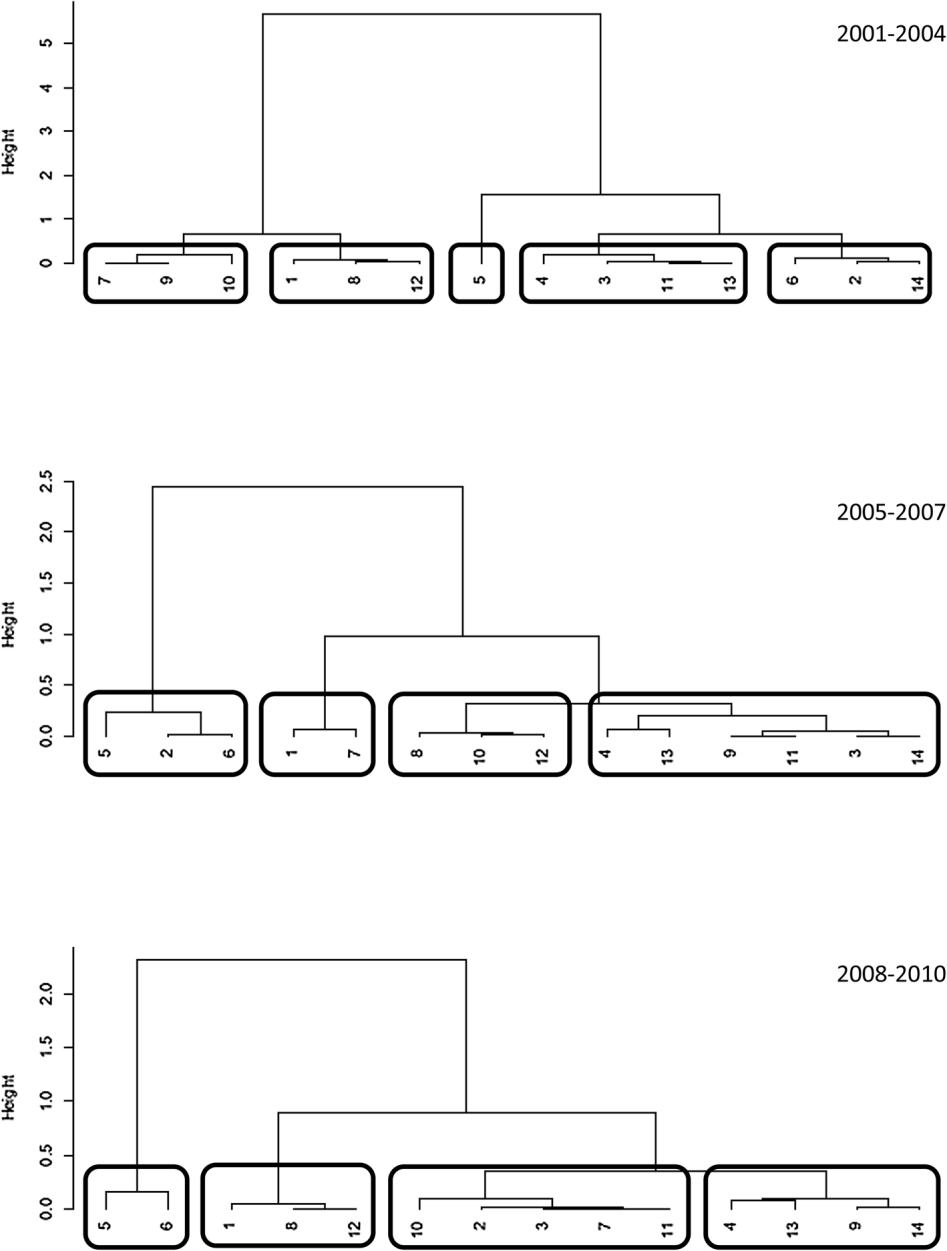
Ward clustering: 2001–2004; 2005–2007; 2008–2010

**Fig. 10:**
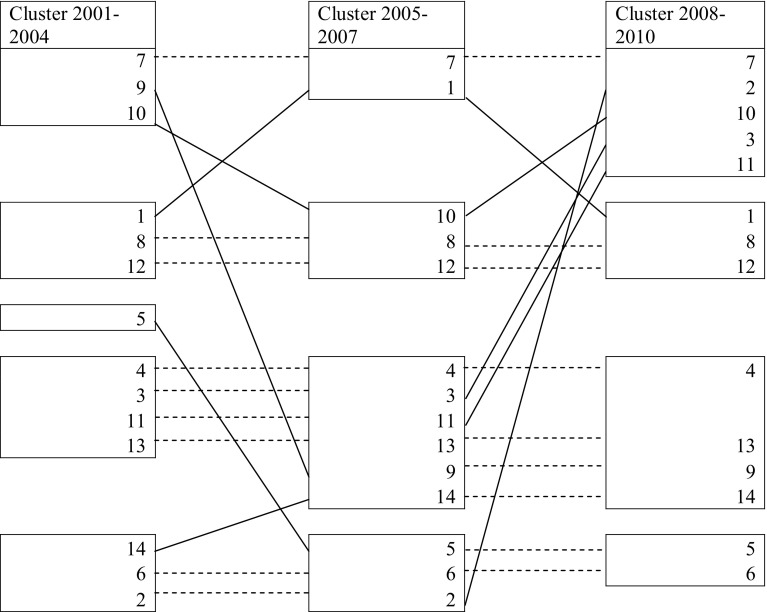
Changes and stability in clusters over time. *Dotted lines* represent no changes, *solid lines* represent changes

We then re-clustered disciplines according to the similarities of their research collaborations, but this time separating each time slice. Results are illustrated in Fig. 9, while changes in clusters summarised in Fig. 10. The longitudinal patterns of collaboration are slightly more complicated to describe, as they do not show very stable tendencies. The most stable cluster is the one that includes 5. Biology and 6. Medicine, that merges in 2005–2007, although in the first two time slots it also adds 2. Physics and 14. Political and Social Sciences. While 14. Political and Social sciences subsequently stabilises in the cluster that includes 13. Economics and statistics and 4. Environmental sciences, 2. Physics shows a more erratic pattern, merging with 6. Medicine in the first two time slots, then with different disciplines both in the last time slot and in the overall clustering procedure.

1. Mathematics and informatics, 12. Law studies and 8. Architecture and civil engineering are included in another relatively stable cluster, although in the second time period 1. Mathematics merges with 7. Agriculture and veterinary, while 12. Law studies and 8. Architecture and civil engineering add 10. Ancient studies. The three original disciplines come together again in the last period.

7. Agriculture and veterinary, 3. Chemistry and 11. History, philosophy, pedagogy and psychology sciences merge in a single cluster in the last period, and the similarities are detected also in the single solution clustering procedure. The cluster also included 9. Industrial and information engineer at the beginning, while in the last period it added 2. Physics and 10. Ancient studies.

Finally, 4. Environmental sciences, 13. Economics and statistics and 14. Political and social sciences represent another stable cluster (although the latter was added in the second time period), but it also includes other fluctuating disciplines: 9. Industrial and information engineering was added in the last two periods, and 3. Chemistry and 11. History, philosophy, pedagogy and psychology sciences were included in the first two periods. As we have seen, 3. Chemistry and 11. History, philosophy, pedagogy and psychology sciences merge together in the overall clustering procedure.

The final part of the analysis looks at the overall amount of funding of sub-disciplinary, in-disciplinary and out-disciplinary research over time. Table 5 summarises the total amount of money funded in each cluster, and shows that the most funded Cluster is Cluster 1, with a mixed profile in terms of disciplinary versus interdisciplinary collaboration; followed by Cluster 3 which is the most interdisciplinary cluster; Cluster 2 which is the most sub-disciplinary cluster, and finally Cluster 4 dedicated to in-disciplinary research. As we mentioned before, the funding allocation depends also on research costs, which tend to be higher in experimental disciplines like Physics, Biology, Medicine, Agriculture and veterinary, Chemistry, but this is not directly reflected in the amount of funding: Cluster, 4, which includes Physics, is the one that receives less money, although Cluster 1 and 3, which include most of the experimental disciplines, do receive most of the funding. We cannot control for the detailed costs or research budgets, so care should be taken in interpreting results.

**Table 5:** Distribution of funding across the 4 clusters

	Total amount of funded money (€)
Cluster 1	388,867,527
Cluster 2	223,837,612
Cluster 3	382,304,597
Cluster 4	190,171,239

To see if multidisciplinary projects receive more or less funding than collaborations within disciplines, we correlate the amount of money allocated for PRIN projects in all disciplines in each time period with the number of collaborations sub-discipline, in-discipline, and out-discipline within the same period. We take the logarithm of each variable as they are not all and always normally distributed.^5^ Given the fact that both the total amount of funding and the percentage of interdisciplinary projects have diminished over time, we expect interdisciplinary projects to receive less funding, and their share of funding to reduce over time. Table 6 clearly shows that this is not the case. Multi-disciplinary projects always receive more funding than their sub-disciplinary and in-disciplinary counterparts. However while in 2001–2004 and in 2008–2010 the gap between the different types of research is not very wide, it is much larger in 2005–2007, which is also the period in which the overall funding of PRIN projects reduced most. In this period the share of funding of in-disciplinary projects is also lower than the sub-disciplinary one (although the value is not significant), while the relation between the two is reversed in the other time periods.

**Table 6:** Pearson correlations of log values of research collaborations against log values of funded money for each time period

Pearson correlation (sig. 2-tailed)	Logsubdisc	Logindisc	Logoutdisc
2001–2004	0.751**	0.824**	0.913**
Logmoney			
2005–2007	0.606*	0.498	0.861**
Logmoney			
2008–2010	0.742**	0.783**	0.846**
Logmoney			

* *p* < 0.05; ** *p* < 0.01

## Discussion

We can now go back to our research questions and discuss the answers that our data analyses suggest. We started by asking how much interdisciplinary research has been effectively conducted in Italy from 2001 to 2010. Looking at the distribution of research collaboration across all disciplines and for all the years under analysis, we can conclude that overall interdisciplinarity only counts for a minor part of academic research. This is true especially for research that cut across disciplinary areas, and that counts for 11 % of all the collaborations. But it is also true for research that includes different sub-disciplines within the same disciplinary area, that overall reaches 17 % of collaborations. The majority of collaboration (72 %) is established within the same sub-discipline, and this percentage increases along the years. On the contrary, in-disciplinary and out-disciplinary research decreases over time, although it slightly rises again for some disciplines in the last period under analysis (2008–2010). This tendency seems to follow the reduction of MIUR funding for PRIN projects, which is drastic in 2006: when the funding are at their lowest, research withdrawn within the more familiar area of disciplinary collaboration. When funding increase again after 2007, some disciplines go back in investing in interdisciplinary research. This is especially true for the cluster of disciplines that mostly tend to invest in subdisciplinary research (Cluster 2), which in the last period slightly increase both in-discipline and out-discipline research. Cluster 3, which invests more in out-discipline research, increases out-disciplinary collaboration when funding became more available, while Cluster 4 increases in-discipline research, which is its own characteristic.

The clustering procedure allowed us to merge together disciplinary areas which show similar patterns in research collaboration. Results do not offer a linear and robust narrative, and they do not indicate a clear cut between hard and soft sciences, which is usually proposed to explain differences in research organizations. Clusters seem to suggest a tendency for disciplines to organise around epistemological rather than ontological dimensions. In other words, if we are to find a common ground for disciplines in clusters, it seems that similarities relate to the methods of investigations rather that the topics of research. Cluster 1, with a mixed profile of collaborations, includes disciplines dedicated to experimental (Chemistry, Psychology, Pedagogy) or applied research (Engineering, Agriculture and Veterinary) but also outliers like History and Philosophy. Cluster 2, dedicated to sub-disciplinary research, includes all non-experimental disciplines, based on theoretical modelling and calculus (Mathematics and information science; Architecture and civil engineer) or on archival data (Ancient studies, Literature and Philology, History and Art; Law studies). Cluster 3, which has the highest interdisciplinary tendencies, merges together clinical research (Biology and Medicine), while Cluster 4, which focuses on in-disciplinary research, includes disciplines that deal with the human and natural environment and that are based on primary data collection and empirical observation (with Physics as an outlier). This is not a surprise, to a certain extent, if we think that one of the main barriers that scholars face when collaborating to interdisciplinary research are epistemic barriers that involve “incompatible styles of thought, research traditions, techniques, and language that are difficult to translate across disciplinary domains” (Jacobs and Frickel 2009: 47). Integration seems easier in what Klein (2008: 17) calls the “middle-range and narrow gauged or horizontal forms of interdisciplinarity among neighbouring disciplines with compatible epistemologies”, rather than among disciplines with more divergent epistemologies.

Furthermore, the longitudinal clustering procedure seems to suggest an overall instability in the evolution of disciplinary similarities, although as we have seen it is possible to identify some relatively stable bundles of disciplines. Biology and Medicine are quite stable and highly interdisciplinary, indicating a robust tendency of these two disciplines. Mathematics and informatics, Law studies and Architecture and civil engineer also form a relatively stable cluster, like Environmental sciences, Economics and statistics and Political and social sciences. Other disciplines exhibit more erratic behaviours, and therefore it is difficult to interpret results.

These differences suggest a certain consistency with the distinction proposed by the literature between theory driven disciplinary research and application oriented interdisciplinary research (Gibbons et al. 1994). In our case it seems that more theoretical disciplines tend to concentrate less in interdisciplinary research than their experimental and empirical counterparts. Cluster 2, characterised by sub-disciplinary research, includes disciplines like Maths, Juridical studies and Ancient Studies which are mostly dedicated to theoretical reasoning. Cluster 4, characterised by in-disciplinary research, includes disciplines like Physics, Environmental sciences, Economics and Political and social sciences which are more applicative. Cluster 3, characterised by out-disciplinary research, includes disciplines like Medicine and Biology which are highly applicative and problem oriented. As we said, Cluster 1 is of difficult explanation given the lack of clear tendency toward disciplinary or interdisciplinary research.

What seems to be clear however is that interdisciplinary research receives overall more funding than their sub-disciplinary and in-disciplinary counterparts. The results of correlations between the number of sub-disciplinary, in-disciplinary and out-disciplinary collaborations against the total amount of money funded over time indicate that despite the increasing reduction of funding, interdisciplinary research receive more money, especially in periods when the budget is drastically reshaped. The strategy of withdrawing within the familiar walls of disciplinary research when funding decrease is not a successful one, as interdisciplinarity still provides more money. Ideally, we should control for the effective costs of research, to see if more expensive research correlates with allocation of funding, but our data do not provide the necessary information.

Our results are partially in line with some previous disciplinary classifications. Boyack et al. (2005) for example, in their map of co-citations within and across disciplines, find that most of Social sciences are independent (thus more disciplinary based), followed by Psychology, Physics, Chemistry and Earth Sciences, which are less disciplinary based, but not as interdisciplinary as Medicine and Biochemistry. Our data confirm the high interdsiciplinarity of Medicine, Biology and Chemistry, but do not show the disciplinary tendencies of Social sciences, which on the contrary reasonably invest in research that cut across disciplinary sectors and areas. Kronegger et al. (2015) find that Mathematics and Landscape design (that in our classification is incorporated in the discipline of Architecture and civil engineering) are clustered with Social sciences and Humanities. In our clustering procedure (single solution) they are merged with Ancient studies and Law studies, confirming the behavioural similarities with Humanities. Qin et al. (1997) similarly find a high level of interdisciplinarity in articles’ citations of Medicine, Biology, Chemistry, Physics and Engineering, and a low level for Mathematics, thus in line with our finding, although they do not analyse Humanities and Social sciences and therefore can only offer partial comparison. In line with our finding, although not completely comparable, is also the study of Morillo et al. (2003), which find a high level of interdisciplinarity for Biomedicine and Technology, and a low level for Humanities, with Mathematics appearing as the less interdisciplinary area of hard sciences.

## Conclusions

The analysis of scientific collaboration within and across academic disciplines is an important area of research for the social studies of sciences. While many researchers concentrate on the analysis of co-authorship and citation, in this article we complement those efforts by looking at collaboration to research projects in Italian Academia. The empirical analysis of this type of collaboration offers an additional perspective over the patterns of disciplinary and interdisciplinary research, showing how the former is still dominant over the latter in Italian Academia, and how interdisciplinarity suffers from the decline of public funding. However, our data also show that interdisciplinary research is overall more remunerative than its disciplinary counterpart, as even in a period of funding reduction it still attracts higher research budgets. Also, interdisciplinary patterns are different across different disciplines, in line with previous results obtained from the analysis of co-authorship and citations networks. These results are not only important for the advancement of social studies of sciences, but can also have a potential impact on research policies and academic organizations. Funding bodies who wish to stimulate interdisciplinary research should be aware of the risk of reducing funding budgets, as this may push scientist to withdraw within the familiar and more mainstream boundaries of their disciplinary areas. Universities and research centres managers could benefit from the observation that interdisciplinary research seems to receive more funding even in times of austerity, while researchers should be encouraged to look beyond their disciplines and establish connections with other areas.

Our results obviously lack the power of generalizability to other geographical areas; further research should be addressed toward comparative studies not only across countries, but also between national contests and, for example, the European Union, and across various continents. It would be interesting to compare research collaborations in US and EU, but also in emerging scientific contexts like South-America or China. Given the fact that data for most public lines of funding are available online this area of research is undoubtedly promising. Also, like all the studies in bibliometrics and scientometrics, our study relies on the pure quantification of research relations, without having the opportunity to explore the effective meaning of such relations. While collaborations to research projects can be considered, to a certain extent, a good proxy for effective scientific cooperation, the mere fact of being named in a research project does not say much about the nature of the cooperation. Further research should be directed toward more qualitative/ethnographical and/or survey approaches, like the one of Cummings and Kiesler (2005) or Rhoten (2004), where scientists can elaborate on their personal ties and discuss the subjective meanings of these ties and their evolution over time. In particular, it would be interesting to understand what effectively a research collaboration involve, and how people perceive disciplinary and interdisciplinary research; how they effectively react to variations in research funding, and if they are aware of the structural effects of funding reductions over interdisciplinary collaboration.

As we have already discussed, our data are also limited in time, therefore calling for longer timescales in order to investigate the modifications in the trends of collaboration. Longer periods can provide more robust results in the dynamics of disciplinary clusters, and could link them to the wider political and cultural scenario of a specific national context. Information on unsuccessful projects could also provide a stronger rationale for the factors of success of research projects. In particular, it would be interesting to observe the ratio of success of either disciplinary bounded and interdisciplinary projects which could reinforce or challenge the observed better performance of the latter. Finally, a fruitful area of research could link the collaboration to research projects with the outputs of such projects, and measure the correlation between cooperation in a project and co-authorship and co-citations. This could measure the reliability of co-authorship and citation data, and the effective productivity of disciplinary and interdisciplinary research. Following this line, multi-mode networks where different types of socio-technical object are measured at the same time represent a promising area of investigation.

## Footnotes

1 Our data are still based on the pre-2008 classification system, with 14 macro-discipline areas and 370 scientific-disciplinary sectors (SDD), to guarantee comparability across the years.

2 The percentage indicates, in each discipline and for each year, the number of projects funded over the number of projects presented. Data are aggregated for Natural and Technical Sciences versus Humanities and Social Sciences, therefore the two values together do not add up to 100 %, as they indicate the rate of success in each macro area respectively.

3 A bipartite network is a specific type of network whose nodes are divided into two sets *X* and *Y*, and only connections between two nodes in different sets are allowed (Breiger 1974).

4 11 of these are ghost people, for whom we could not find any information in the Miur database. We therefore excluded them from the final analysis which is based on 19,442 scientists.

5 Shapiro–Wilk test is significant (*p* < 0.05) for the variables indisc, outdisc and money in 2005–2007 and 2008–2010; non-significant (*p* > 0.05) for subdisc across all time periods and money in 2001–2004.
